# Effects of thickened carbonated cola in older patients with dysphagia

**DOI:** 10.1038/s41598-022-25926-4

**Published:** 2022-12-22

**Authors:** Akino Saiki, Kanako Yoshimi, Kazuharu Nakagawa, Yuki Nagasawa, Akira Yoshizawa, Ryosuke Yanagida, Kohei Yamaguchi, Ayako Nakane, Keisuke Maeda, Haruka Tohara

**Affiliations:** 1 Department of Dysphagia Rehabilitation, Division of Gerontology and Gerodontology, Tokyo Medical and Dental University, Tokyo, Japan; 2 Department of Geriatric Medicine, National Center for Geriatrics and Gerontology, 7-430 Moriokamachi, Obu, Aichi 474-8511 Japan

**Keywords:** Geriatrics, Quality of life

## Abstract

Carbonated beverages initiate the swallowing reflex earlier than water and have a shorter pharyngeal transit time. However, the effects of carbonation in thickened beverages of the same flavor on swallowing dynamics have not been reported. Therefore, we investigated the effects of thickened carbonated beverages on swallowing in patients with dysphagia by comparing the swallowing dynamics between thickened carbonated and thickened non-carbonated beverages. We enrolled 38 patients with dysphagia and divided them into two groups. Thickened carbonated and thickened non-carbonated beverages were used. Videoendoscopic swallowing evaluations were performed. Aspiration, penetration, pharyngeal residue, and initiation position of the swallowing reflex were evaluated. The reduction in the amount of residue in both the vallecula (*p* = 0.007) and pyriform sinus (*p* = 0.004) was greater after ingestion of thickened carbonated cola than thickened non-carbonated cola. The onset of the swallowing reflex was significantly earlier after ingestion of thickened carbonated cola than thickened non-carbonated cola (*p* = 0.007). There were no significant differences in the extent of penetration. Thickened carbonated beverages positively affected swallowing compared with thickened non-carbonated beverages. Thus, the use of thickened carbonated beverages may be helpful for patients with dysphagia.

## Introduction

The number of patients with dysphagia is expected to increase as the population ages Hence, identifying ways to improve the quality of life of these patients is important. Additionally, appropriate food preparations and thickening of beverages may be required depending on the severity of dysphagia. Patients with dysphagia often experience aspiration when consuming thin liquids^1^. When water is swallowed, it flows quickly through the pharynx, potentially leading to aspiration when deficits in swallowing function exist. To prevent aspiration, liquids can be thickened if other methods are ineffective. The speed of the bolus flowing into the pharynx can be decreased by thickening, thereby preventing aspiration caused by the delay in timing of swallowing^2–6^. Although viscosity varies with the type of beverage and thickener, studies in chronic post-stroke patients suggest that 150–800 mPa·s is an appropriate viscosity for patients with dysphagia. Meanwhile, 250 mPa·s is the minimum viscosity that may prevent aspiration, whereas a viscosity above 800 mPa·s is still safe^7,8^.

Additionally, the stimulation from food itself affects swallowing dynamics. Transient receptor potential agonists, such as capsaicin, menthol, or the olfactory stimulation of black pepper, promote the initiation of the swallowing reflex^9^. Recently, smaller amounts of carbonated water initiate the swallowing reflex earlier than water in healthy adults^10^. Furthermore, the pharyngeal transit time is shorter in patients with dementia and Parkinson’s disease when carbonated water is consumed vs. plain or thickened water^11^. The posterior wall of the pharynx and the anterior and posterior faucial pillars are sensitive to chemical stimuli that initiate swallowing reflexes^12^. These stimuli are transmitted to the swallowing center via the pharyngeal branches of the glossopharyngeal and superior laryngeal nerves^13^. Chemical stimulation due to the effervescence of carbonic acid stimulates sensory receptors and activates innervating nerves to promote swallowing^14–16^. Carbonated liquids improve swallowing, which may prevent penetration and aspiration^16^. The effect of thickened carbonated beverages on swallowing dynamics has been verified in previous studies^17^. However, the effects of carbonation in commercially available thickened carbonated beverages have not been explored. Therefore, this study investigated the effects of carbonation in thickened carbonated beverages by comparing the swallowing dynamics with and without carbonation.

## Results

### Line-spread test

The flow distance of Line-Spread Test (LST) was 3.42 cm in thickened carbonated cola, and 3.54 cm in thickened non-carbonated cola. Although LST values do not precisely correlate with viscosity, both thickening concentrations were estimated to be moderately thick based on the Japanese Dysphagia Diet 2021.

### Participant characteristics

The participants’ characteristics are shown in Table 1. Overall, 38 participants were included in the study; 19 were men, and the patients had median age of 80 (74–85) years. The primary diseases included organic disease (23.7%), cerebrovascular disease (21.1%), neurological disease (21.1%), dementia (15.8%), and others (18.4%).

**Table 1:** Patient characteristics.

**Sex**	
Male (N, %)	19 (50.0)
Female (N, %)	19 (50.0)
Age (years) (median, IQR)	80 (74, 85)
**Primary disease of dysphagia (N, %)**	
Organic disease	9 (23.7)
Cerebrovascular disease	8 (21.1)
Neurological disease	8 (21.1)
Dementia	6 (15.8)
Others	7 (18.4)
**DSS (N, %)**	
1	0 (0)
2	4 (10.5)
3	15 (39.5)
4	8 (21.1)
5	6 (15.8)
6	5 (13.2)
7	0 (0)
**FOIS (N, %)**	
1	0 (0)
2	12 (31.6)
3	5 (13.2)
4	3 (7.9)
5	9 (23.7)
6	9 (23.7)
7	0 (0)

*N* number, *IQR* interquartile range, *DSS* dysphagia severity scale, *FOIS* functional oral intake scale.

### Swallowing dynamics

**Figure 1:**
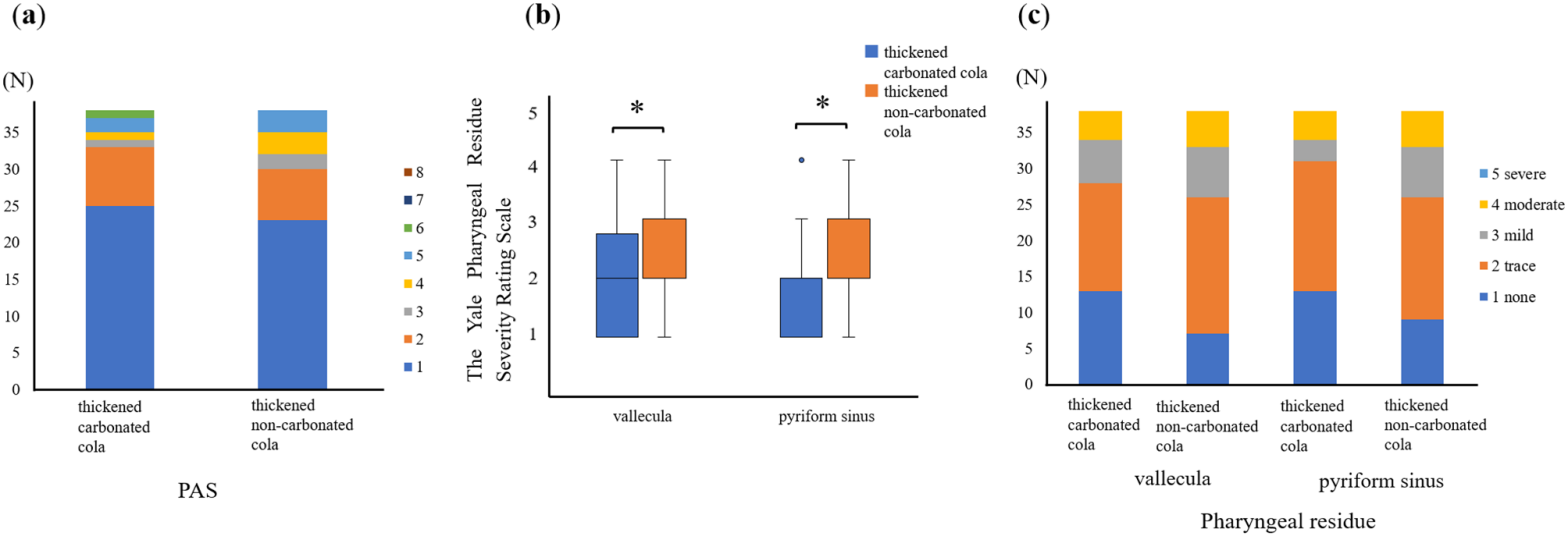
Comparison of swallowing dynamics between thickened carbonated and thickened non-carbonated cola. (**a**) Comparison of penetration/aspiration with thickened carbonated cola and thickened non-carbonated cola. (**b**) Boxplot comparing the amount of pharyngeal residue between thickened carbonated cola and thickened non-carbonated cola (*p < 0.05). (**c**) Bar graph comparing the amount of pharyngeal residue for thickened carbonated cola and thickened non-carbonated cola. *PAS* Penetration-Aspiration Scale.

**Figure 2:**
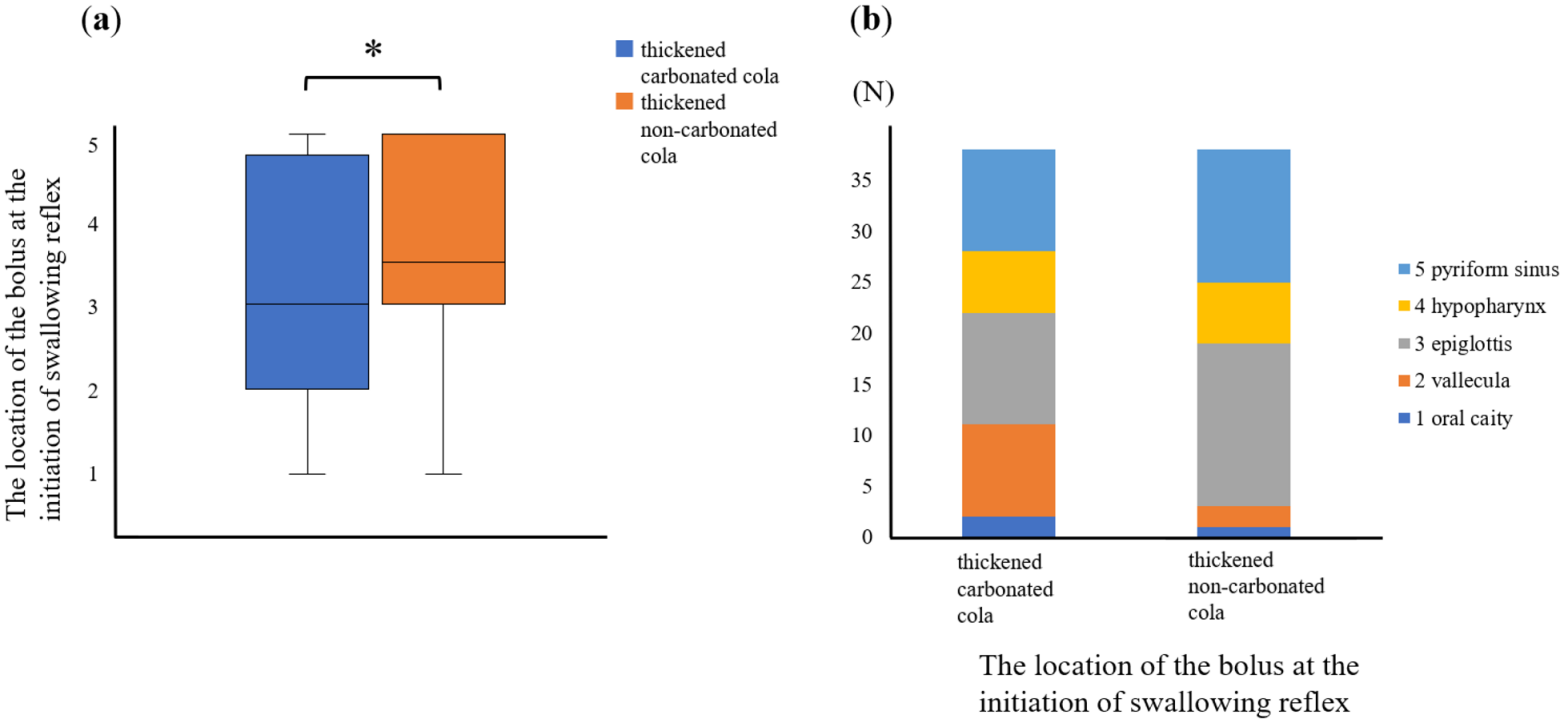
Comparison of the location of bolus at the initiation of swallowing between thickened carbonated cola and thickened non-carbonated cola (**a**) box plot; *p < 0.05, (**b**) bar chart.

The results of the Wilcoxon signed-rank test are summarized in Table 2. There was no significant difference in the extent of penetration and aspiration between thickened carbonated cola (1 [1–2]) and thickened non-carbonated cola (1 [1–2]) (*p* = 0.271) as shown by the Penetration-Aspiration Scale (PAS) (Fig. 1a). Thickened carbonated cola (2 [1–2.75]) resulted in significantly less pharyngeal residue in the vallecula than thickened non-carbonated cola (2 [2–3]; *p* = 0.007) (Fig. 1b, c). Thickened carbonated cola (2 [1–2]) also resulted in significantly less pharyngeal residue in the pyriform sinus than thickened non-carbonated cola (2 [2–3]; *p* = 0.004) (Fig. 1b, c). Additionally, thickened carbonated cola initiated the swallowing reflex significantly earlier (3 [2–4.75]) than thickened non-carbonated cola (3.5 [3–5]; *p* = 0.007) (Fig. 2a, b).

**Table 2:** Comparison of thickened carbonated and non-carbonated cola.

	Thickened carbonated cola		Thickened non-carbonated cola		*p *value
	Median (IQR)	Min, Max	Median (IQR)	Min, Max	
PAS score	1 (1–2)	1, 6	1 (1–2)	1, 5	0.271
Pharyngeal residue (vallecula)	2 (1–2.75)	1, 4	2 (2–3)	1, 4	0.007*
Pharyngeal residue (pyriform sinus)	2 (1–2)	1, 4	2 (2–3)	1, 4	0.004*
Location of the bolus at the initiation of swallowing reflex	3 (2–4.75)	1, 5	3.5 (3–5)	1, 5	0.007*

Penetration-Aspiration Scale.

1: contrast does not enter the airway, 2: contrast enters the airway; remains above vocal folds, no residue, 3: contrast remains above vocal folds, visible residue remains, 4: contrast contacts vocal folds, no residue, 5: contrast contacts vocal folds, visible residue remains, 6: contrast passes glottis, no subglottic residue visible, 7: contrast passes glottis, visible subglottic residue despite patient’s response, 8: contrast passes glottis, visible subglottic residue, absent patient response.

The Yale Pharyngeal Residue Severity Rating Scale (vallecula).

1: none; no residue, 2: trace; trace coating of the mucosa, 3: mild; epiglottic ligament visible, 4: moderate; epiglottic ligament covered, 5: severe; filled to epiglottic rim.

The Yale Pharyngeal Residue Severity Rating Scale (pyriform sinus).

1: none; no residue, 2: trace; trace coating of mucosa, 3: mild; up wall to quarter full, 4: moderate; up wall to half full, 5: severe; filled to aryepiglottic fold.

Location of the bolus at the initiation of swallowing reflex.

1: oral, 2: vallecula, 3: epiglottis, 4: hypopharynx, 5: pyriform sinus.

*IQR* interquartile range, *PAS* Penetration-Aspiration Scale.

*p values < 0.05 are significant.

## Discussion

This study showed that thickened carbonated cola positively affects the swallowing dynamics of patients with dysphagia compared with thickened non-carbonated cola. The study participants were older adults who had a variety of diseases and varied severities of dysphagia. This study also showed that consumption of thickened carbonated cola resulted in less pharyngeal residue than thickened non-carbonated cola.

Sensory stimuli are essential to swallowing. Various oral stimuli, such as nociception, chemical stimuli, and pain and tactile stimuli including air pulses, cause swallowing movements^12,18,19^. The posterior wall of the pharynx and the anterior and posterior faucial pillars are sensitive areas for chemical stimuli that initiate swallowing reflexes^12^. Furthermore, sensory stimuli near the vocal cords effectively induce the swallowing reflex^13,20^. The trigeminal, facial, glossopharyngeal, and superior laryngeal nerves are mainly involved in afferent sensory inputs related to swallowing movements^15^. Chemical stimuli induced by effervescence from carbonic acid stimulate pain perception in areas sensitive to stimuli that induce the swallowing reflex, which is innervated by the glossopharyngeal and pharyngeal branches of the superior laryngeal nerve and induces afferent sensory input^14,15,21^.

Additionally, carbon dioxide in carbonated beverages is converted to carbonic acid in the oral cavity, which stimulates nociceptors in the tongue^22,23^. Sensory stimuli in the oral and pharyngeal regions are transmitted via afferent nerves to the brain, where they activate swallowing mechanisms^19^. The present study indicates that sensory stimulation by carbonic acid on the motor systems associated with swallowing may have led to the early initiation of the swallowing reflex and a reduction of pharyngeal residues.

Swallowing movements are affected by taste and chemical stimuli^24^. Acidic stimuli in the oral and pharyngo-laryngeal regions induce swallowing and improve the timing of swallowing initiation. Furthermore, acidic stimuli cause strong contractions of swallowing-related muscles, promoting swallowing^25–27^. Although the swallowed volume of carbonated water was less than that of plain water, the masseter and suprahyoid muscles more strongly contracted with carbonated water compared to plain water^28^. A study using rats reported that acid taste receptors in the oral cavity react with carbon dioxide dissolved in carbonated beverages^29^. Thickened carbonated beverages are effective for swallowing in patients with pharyngeal phase disorders; however, caution should be taken when giving these beverages to patients with dysphagia who have oral phase disorders, such as difficulty feeding food into the pharynx, due to the risk of teeth decalcification or erosion of the oral cavity’s residuals. The ingredients of cola include sugar, carbonic acid, caramel color, acidulants, and flavorings, and it has a pH of approximately 2.4. The critical pH at which teeth demineralize is approximately 5.5; hence, consumption of thickened cola increases tooth demineralization or erosion^30^. If the oral cavity is acidic, the amount of wear caused by brushing is greater than if it is neutral^31^. Therefore, while the acidity is neutralized by the buffering capacity of saliva^32^, oral care, including cleaning the mouth with a sponge brush or gauze after consuming thickened carbonated beverages, is necessary to prevent thickened carbonated beverages from remaining in the oral cavity.

Carbonated beverages are very popular worldwide. However, patients with dysphagia who have oropharyngeal disorders often have difficulty drinking liquids due to the risk of aspiration. This study allowed patients with dysphagia who favored carbonated beverages but could not consume them due to the risk of aspiration to taste thickened carbonated beverages. The positive effects of thickened carbonated beverages on swallowing can also be used in the rehabilitation of patients with dysphagia. For example, patients with dysphagia who have decreased pharyngeal contraction due to aging or disuse and are at a high risk of aspiration due to pharyngeal residues, may be able to reduce their risk of aspiration by consuming thickened carbonated beverages, which are less likely to have pharyngeal residues as compared to thickened non-carbonated beverages. In some cases, inducing swallowing is difficult due to a decline in oral function caused by a prolonged lack of oral intake or decreased level of consciousness or physical function. In such cases, using thickened carbonated beverages would be an effective strategy in the rehabilitation of patients with dysphagia with a low risk of aspiration. The taste and effervescent stimulus of thickened carbonated beverages can also help activate the oral, pharyngeal, and laryngeal senses. Furthermore, dysphagia can be caused by decreased muscle strength as well as a decrease in sensory function due to aging or neurological and anatomical factors^2,3,33,34^. Therefore, compensatory strategies through sensory stimulation using food, such as carbonated drinks, is considered effective for dysphagia rehabilitation^35^.

Our study had several limitations. First, since we did not compare thickened carbonated beverages with non-thickened carbonated beverages, it was difficult to determine whether the effect of carbonation in thickened carbonated beverages was equivalent to that of non-thickened carbonated beverages. Moreover, since the same product was compared, it was thought that there was no difference in the initial viscosity of the beverage. However, the effects of the differences in the ingredients and thickening products on the physical properties of thickened carbonated beverages and swallowing must be considered. Second, the appropriate thickening concentration varies depending on the severity of dysphagia; therefore, it is necessary to determine whether a change in the degree of thickening would result in a change in swallowing dynamics. The food thickener used in this study was xanthan gum-based. Third, the effervescence of thickened carbonated beverages makes it difficult to accurately measure gas volume. Therefore, gas volume in thickened cola was not measured.

Commercially available carbonated beverages can be easily and conveniently consumed by anyone. Meanwhile, patients with dysphagia who require thickened beverage may benefit from the effects of carbonic acid for swallowing training. In the future, we plan to perform an interventional study to investigate whether compensatory strategies using thickened carbonated beverages will improve the effect of approaches in hospitalized patients with dysphagia.

## Conclusion

This study showed that thickened carbonated beverages positively affect swallowing in patients with dysphagia because thickened carbonated beverages reduce pharyngeal residue and induce the swallowing reflex earlier than thickened non-carbonated beverages.

## Methods

### Trial design and participants

This crossover randomized controlled study was conducted at the Tokyo Medical and Dental University Hospital (Tokyo, Japan) and confirmed with the CONSORT statement. A total of 38 patients with dysphagia who needed videoendoscopic swallowing evaluation at the Department of Dysphagia Rehabilitation of Tokyo Medical and Dental University in October 2022 were enrolled. Patients were diagnosed with dysphagia based on swallowing function assessment, including videoendoscopic examinations (VE) or video fluoroscopic examinations prior to the study’s implementation. The exclusion criteria were those who had difficulties during swallowing evaluation with severe aspiration, significant medical disorders, functional oral intake scale (FOIS)^36^ score of 7, and non-consent to enrollment. This study was approved by the Dental Research Ethics Committee of Tokyo Medical and Dental University (D2020-047) and was registered in the UMIN clinical trial registration system on September 3, 2020 (UMIN000041674). All procedures performed were in accordance with the ethical standards of the institutional and/or national research committee and with the 1964 Helsinki Declaration and its later amendments or comparable ethical standards. Informed written informed consent was obtained from all participants or their legal representatives.

### Randomization and masking

We conducted a two-group comparison of the swallowing dynamics between thickened carbonated and thickened non-carbonated beverages. To eliminate potential order effects from swallowing each test liquid, participants were randomly divided into two groups using the numbered container method: those who first swallowed thickened carbonated beverages and those who swallowed thickened non-carbonated beverages. The participants were blinded to the order in which they swallowed the test liquid.

### Procedure

Thickened carbonated beverages used in the study were prepared the day before the test by a single dentist.

Commercial carbonated beverage colas, which contain approximately 3.7–3.8 GV of carbon dioxide gas, were used as the test liquids because it was familiar and readily available. Food thickener (5 g; Neo-High Toromeal III, FoodCare, Kanagawa, Japan) was added to a 500-mL plastic bottle of cola (1 g/100 mL) and immediately mixed by closing the lid and shaking for about 30 s to create thickened cola. The cola was thickened according to the viscosity of the International Dysphagia Diet Standardization Initiative Level 3 equivalent. The bottle of thickened cola was placed sideways in the refrigerator for 12–18 h before testing started to allow sufficient time for the carbonic acid to dissolve into the liquid. This manner prevented the loss of gas due to the addition of the thickening agent and shaking. Non-carbonated cola was prepared by leaving a bottle of cola with its lid open in the refrigerator for 24 h. Thickened non-carbonated cola was prepared at the time of testing using the same food thickener and the same viscosity. The temperature of the thickened carbonated and thickened non-carbonated beverages was approximately 10 °C. Each test liquid was colored with food coloring to blind the examiner and participants.

The participants’ age, sex, primary disease, dysphagia severity scale^37^, and FOIS^36^ were extracted from the medical records. VE were performed to evaluate the swallowing dynamics when participants swallowed thickened carbonated colas and thickened non-carbonated colas. A fiberoptic endoscope (Pentax Japan, Tokyo, Japan) was inserted through the nostrils of the participants while they were sitting in a chair or lying on a bed. No anesthesia was used to insert the fiberoptic endoscope through the nostrils. Following the order assigned to them, 5 mL of test liquid was placed into their mouth using a syringe, and they were instructed to swallow. This process was repeated after ensuring that the previous liquid had been swallowed. Each test sample was ingested three times consecutively. From the VE data, we evaluated the presence of aspiration or penetration, pharyngeal residue in the vallecula and pyriform sinus, and position of initiation of the swallowing reflex.

### Assessment of swallowing function

Aspiration and penetration were evaluated using the Penetration-Aspiration Scale (PAS)^38^ on a scale of 1 (no penetration and aspiration; contrast does not enter the airway) to 8 (aspiration; contrast passes the glottis, visible subglottic residue, and absent patient response).

The amount of pharyngeal residue in the vallecula and pyriform sinus was evaluated separately using the Yale Pharyngeal Residue Severity Rating Scale^39^ on a scale of 1 (none) to 5 (severe).

The position of the tip of the food bolus at the point of swallowing initiation was classified into five levels: oral cavity, vallecula, epiglottis, hypopharynx, and pyriform sinus^40^.

All evaluations were performed by a single dentist who works at the department of dysphagia rehabilitation in the university hospital and was blinded to the type of cola consumed. The lowest scores of the three ingestions were adopted for each trial.

### Statistical analysis

The Wilcoxon signed-rank test was performed to compare the PAS, the Yale Pharyngeal Residue Severity Rating Scale, and position of swallowing reflex between thickened carbonated and thickened non-carbonated cola. The significance level was set at p < 0.05, and IBM SPSS for Windows, Version 28.0 (IBM Japan, Tokyo, Japan) was used for the statistical analysis. Data are presented as median (interquartile range).

### Sample size calculation

G*power 3.1 (Heinrich Heine University, Duesseldorf, Germany) was used to calculate the number of participants required, which revealed that at least 35 participants were required (power: 0.8, effect size: 0.5, tails: two tailed, parent distribution: normal). Calculations were made with reference to the previous study^41^.

### Line-spread test

Accurate measurement of the viscosity of thickened carbonated beverages with a rotational viscometer is challenging. In this regard, measuring a constant amount of liquid is difficult due to the effect of carbon dioxide gas dissolved in the liquid and the production of carbonic acid bubbles by the measurement technique. Similarly, it is difficult to measure viscosity with a syringe test because air bubbles in the thickened carbonated beverage prevents the liquid from dripping. Therefore, the thickening concentrations of the thickened carbonated beverage and thickened non-carbonated beverage used in this study were measured using LST^42^.

The LST equipment (Saraya, Osaka, Japan) was used. A cylinder (3.0 cm in height and 2.8. cm in diameter) was placed in the center of concentric circles (drawn every 1 mm) on a plastic plate. The cylinder was filled with 20 mL of sample. The temperature of each sample was approximately 10℃. After 30 s, the cylinder was lifted, and the sample was allowed to flow for 30 s. Subsequently, the distance that the sample flowed was measured at 6 points, and the average value was calculated. Each trial was performed 3 times on each test liquid, and the average was calculated.

## Data Availability

The datasets generated and analyzed during the current study are not publicly available due to them containing information that could compromise the privacy of the participants but are available from the corresponding author upon reasonable request.
