# Author Correction: Effects of thickened carbonated cola in older patients with dysphagia

**DOI:** 10.1038/s41598-023-30109-w

**Published:** 2023-02-21

**Authors:** Akino Saiki, Kanako Yoshimi, Kazuharu Nakagawa, Yuki Nagasawa, Akira Yoshizawa, Ryosuke Yanagida, Kohei Yamaguchi, Ayako Nakane, Keisuke Maeda, Haruka Tohara

**Affiliations:** 1 Department of Dysphagia Rehabilitation, Division of Gerontology and Gerodontology, Tokyo Medical and Dental University, Tokyo, Japan; 2 Department of Geriatric Medicine, National Center for Geriatrics and Gerontology, 7-430 Moriokamachi, Obu, Aichi 474-8511 Japan

Correction to: *Scientific Reports* 10.1038/s41598-022-25926-4, published online 22 December 2022

The original version of this Article contained errors.

In Figure 1, the colours in the figure legend of panel (b) were reversed.

Similarly, in Figure 2, the colours in the figure legend of panel (a) were reversed.

The original Figures 1 and 2 and accompanying legends appear below.

**Figure 1:**
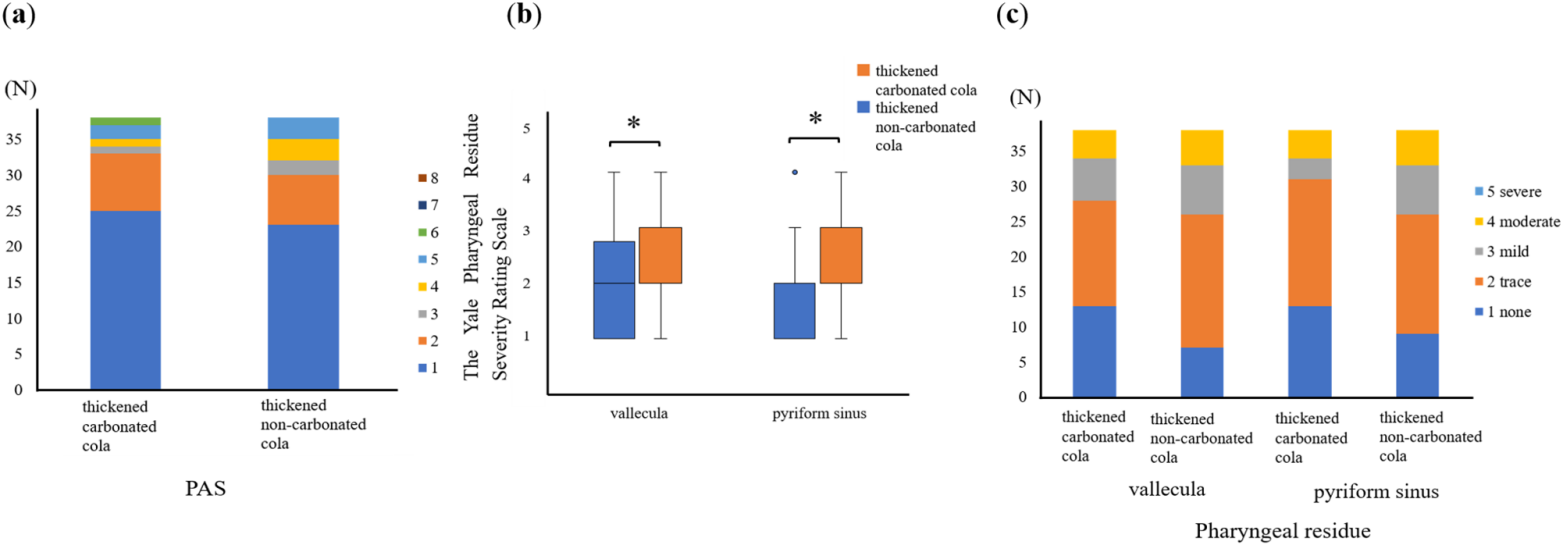
Comparison of swallowing dynamics between thickened carbonated and thickened non-carbonated cola. (**a**) Comparison of penetration/aspiration with thickened carbonated cola and thickened non-carbonated cola. (**b**) Boxplot comparing the amount of pharyngeal residue between thickened carbonated cola and thickened non-carbonated cola (*p < 0.05). (**c**) Bar graph comparing the amount of pharyngeal residue for thickened carbonated cola and thickened non-carbonated cola. *PAS* Penetration-Aspiration Scale.

**Figure 2:**
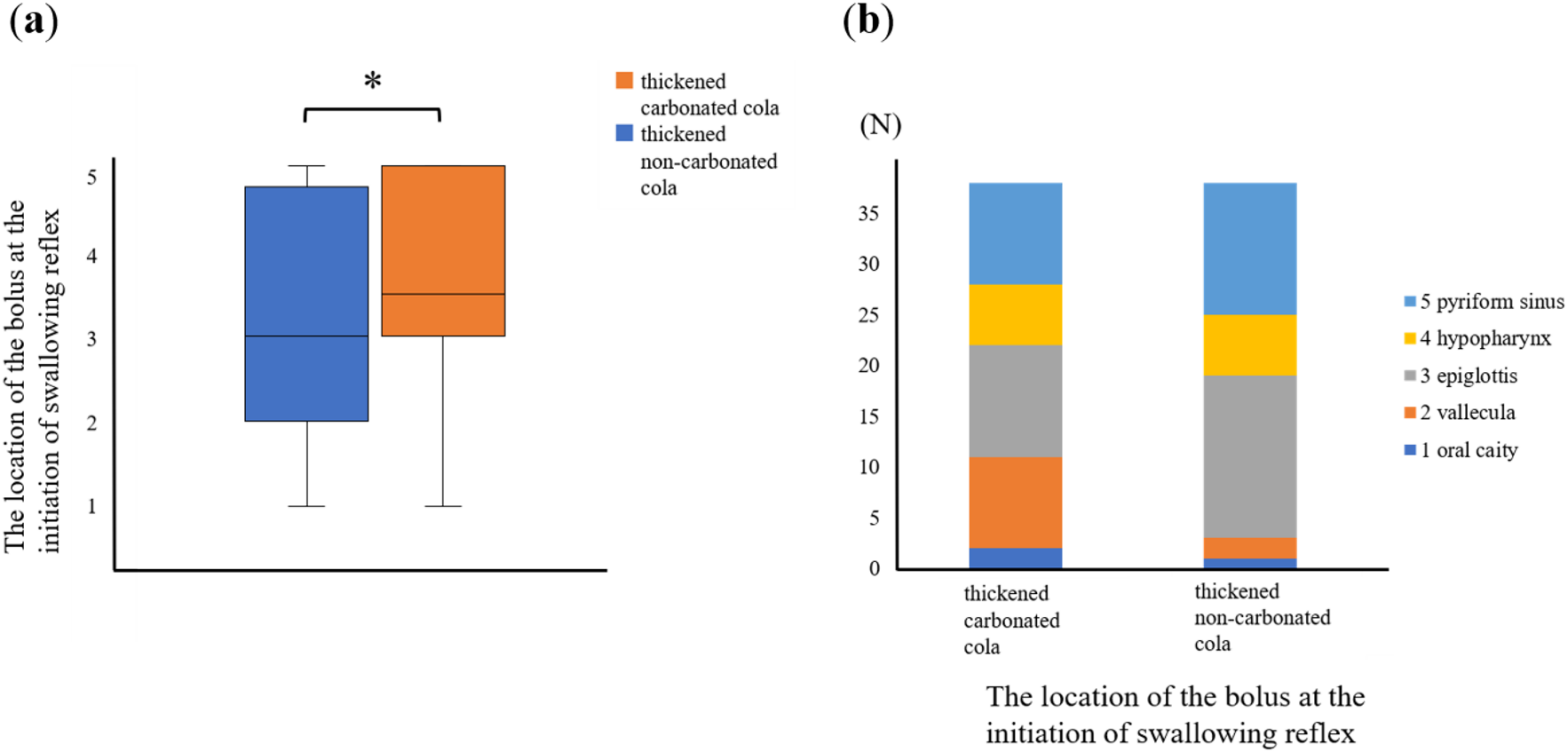
Comparison of the location of bolus at the initiation of swallowing between thickened carbonated cola and thickened non-carbonated cola (**a**) box plot; *p < 0.05, (**b**) bar chart.

The original Article has been corrected.

